# Trends in prevalence and determinants of stunting in Tanzania: an analysis of Tanzania demographic health surveys (1991–2016)

**DOI:** 10.1186/s12937-019-0505-8

**Published:** 2019-12-10

**Authors:** Bruno F. Sunguya, Si Zhu, Rose Mpembeni, Jiayan Huang

**Affiliations:** 1 School of Public Health and Social Sciences, Muhimbili University of Health and Allied Sciences, Dar es Salaam, Tanzania; 2 School of Public Health, Fudan University, Shanghai, China; 3 Key Laboratory of Health Technology Assessment, National Health Commission, Shanghai, China

**Keywords:** Stunting, Demographic and health survey, Child health, Breastfeeding, Malnutrition

## Abstract

**Background:**

Tanzania has made a significant improvement in wasting and underweight indicators. However, stunting has remained persistently higher and varying between regions. We analyzed Tanzania Demographic and Health Survey (TDHS) datasets to examine (i) the trend of stunting over the period of 25 years in Tanzania and (ii) the remaining challenges and factors associated with stunting in the country.

**Methods:**

This secondary data analysis included six TDHS datasets with data of 37,409 under-five children spreading in 1991–1992(*n* = 6587), 1996(*n* = 5437), 1999(*n* = 2556), 2004–05(*n* = 7231), 2009–10(*n* = 6597), and 2015–16(*n* = 9001) conducted in all regions of Tanzania. Variables specific to children and their caregivers were analyzed using SPSS version 22. The variables considered include child anthropometric variables, caregiver’s demographic characteristics and household’s socio-economic factors. We used frequencies and percentages to compare stunting prevalence across the six surveys and chi-square test and three-level hierarchical logistic regression to examine the factors associated with stunting also applying sample weighting as advised by TDHS.

**Results:**

The prevalence of stunting has declined by 30% over the period of 25 years in Tanzania. However, one in three children aged below five years remains stunted with overweight and obesity more than doubled (from 11 to 25%) in the same period among women of reproductive age. The factors associated with stunting included children living in female-headed households (AOR = 1.16, *P* = 0.014), aged 24–35 months (AOR = 1.75, *P* = 0.019), born with low birth weight (AOR = 2.14, *P* < 0.001) and with inconsistent or without breastfeeding (AOR = 3.46, *P* < 0.001 and AOR = 4.29, *P* = 0.001) respectively. The risk of stunting among children living in urban area (AOR = 0.56, *P* < 0.001), with higher caregiver’s education (AOR = 0.56, *P* = 0.018), obese mother (AOR = 0.63, *P* < 0.001), households with highest wealth index (AOR = 0.42, *P* < 0.001), and among girls (AOR = 0.77, *P* < 0.001).

**Conclusions:**

The burden of stunting in Tanzania has declined by 30% in the past 25 years, but still affecting one in every three children. Efforts are needed to increase the pace of stunting decline especially among boys, children in rural areas, from poor, uneducated, and female-headed households, and through improving infant and young feeding practices. Effective and tailored nutrition-sensitive and specific interventions using multisectoral approaches should be considered to address these important determinants.

## Background

More than 165 million children are stunted globally, with low- and middle-income countries bearing the biggest brunt of this burden [1]. Only 14 countries carry 80% of this burden, with Tanzania ranking tenth [2]. Efforts and investments in health have resulted to a steady decline of stunting globally, however, the speed has been slow in Africa, calling for more tailored interventions suitable for each country [2]. Such efforts include strengthening health systems and integrated management of childhood illnesses, immunization and vitamin A supplementation, and advocacy for improving infant and young child feeding practices including exclusive breastfeeding [3]. These nutrition specific interventions have resulted into a rapid decline in acute forms of undernutrition with modest decline of stunting and other chronic undernutrition. In Tanzania for example, the country was able to meet the millennium development target of 14.4% --ahead of deadline in 2015 [4] owing to such interventions. Similar efforts have not resulted in similar results for stunting. More efforts are therefore needed to strengthen nutrition sensitive interventions that can also improve livelihoods and therefore ameliorate determinants of chronic forms of undernutrition such as poverty, food insecurity, education, and other sociodemographic disadvantages [3, 5].

Determinants of stunting extends from pre-conception, through pregnancy to the child’s second birthday [6, 7]. This window of opportunity calls for strengthening maternal nutrition before and after conception, and within the next one thousand days [8]. Evidence have shown that 20% of stunting has its causes originated in the womb [9], and last for life [8, 10, 11]. Stunting at younger age is also linked with poor cognitive development, low IQ, poor school performance, and early deaths owing to other determinants including early onset of non-communicable diseases [12, 13]. Such causes and consequences repeat in subsequent generations escalating lifelong cost to national economy owing to poor human capital, lost opportunity of affected individuals to participate in the national economy, and costing health care [14]. Investing in efforts to alleviate stunting is therefore an effective as much it is an economical intervention [15].

Over the years, Tanzania like other low- and middle-income countries has made strides in economic transformation that resulted from and into improvement of such basic and underlying causes of undernutrition [1, 16]. With efforts to increase enrollment in primary education and beyond, more people including caregivers are educated, delaying to have their first children, and taking part in formal sectors in the national economy [4, 17]. This has positive impact in their own health, nutrition status, newborn health, food security, and therefore their family’s wellbeing [4]. More women are now heading their families as breadwinners than in the past [4, 17]. Such demographic changes may have impact in nutrition and child health. However, data to ascertain this phenomenon have not been analyzed nor has the changes in nutrition landscape and characteristics thereof in Tanzania. This study therefore, aimed to first, examine the trend of stunting over the period of 25 years in Tanzania. Second, it aimed to examine the remaining challenges and factors associated with stunting in the country. We therefore analyzed data collected from six major surveys conducted over the period of 25 years to explain the changes in nutritional landscape characteristics thereof in relation to stunting among children below five years of age.

## Methods

### Study design

This secondary data analysis was conducted on Tanzania Demographic and Health Surveys datasets. A total of 14 surveys have been conducted since 1991 in Tanzania. They include the demographic and health surveys (DHS), AIDS indicator surveys (AIS), Service Provisional Assessment (SPA), Malaria Indicator Surveys (MIS), and Key Indicator Surveys (KIS) [18]. The DHS has variables that can inform panel data for nutritional, maternal, and child indicators that can be analyzed to address the two objectives. We therefore used all the 6 Standard DHS surveys. This nationally representative surveys are conducted every five years in all regions of the country using similar methods and tools by the National Bureau of Statistics (NBS) in collaboration with Monitoring and Evaluation to Assess and Use Results (MEASURE) DHS.

### TDHS sampling of households

The six TDHS employed the random sampling method taking into consideration the population density to collect data from all administrative regions of the country. Data of 41297 children were available and spread across the years as follows: 1991-92 (7287), 1996 (6080), 1999 (2839), 2004-05 (7852), 2009-10 (7526), and 2015-16 (9713). Not all data had nutrition related variables. We managed to extract data of total of 37409 children under five with nutrition variables, spreading across the years as follows: 1991-92 (6587), 1996 (5437), 1999 (2556), 2004-05 (7231), 2009-10 (6597), and 2015-16 (9001).

### Datasets and variables used for this analysis

We chose to analyze data from six Tanzania Demographic and Health Surveys (TDHS) conducted in 1991–92, 1996, 1999, 2004–05, 2009–10, and 2015–16 because they had variables needed for analysis. To enable comparability of variables, we chose panel variables whose data were collected in similar the manner. Such variables included the demographic characteristics of the care givers, nutritional status of children, household wealth index, and feeding practices.

The main outcome of interest was stunting, measured as height-for-age below -2sd of the given standard population. Severe form of stunting was defined as height-for-age below -3sd of a standard population. The TDHS surveys conducted before 2006 used the CDC standard growth references which were derived from the NCHS/FELS/CDC reference population. This was changed in 2006 when WHO conducted a study on growth (https://www.who.int/childgrowth/en/). To harmonize the survey data, we re-calculated z-scores of the TDHS 1991–1992, 1996, 1999, and 2004–2005 surveys into the new WHO Child Growth Standards, using a syntax file provided by the WHO (http://www.who.int/childgrowth/software/en/).

The independent variables from the child questionnaire included child age in months, child’s sex, birth weight in grams and duration of breastfeeding (months). Child birth weight below 2500 g was considered low birth weight while 2500-4000 g was normal and above which was considered big baby. Independent variables pertinent to caregivers were extracted from the women’s questionnaire. They included caregiver’s age (years), highest education level (primary, secondary or higher), age at first child birth (years), number of children living in their households, and place of residence (urban or rural). Other variables included the sex of the household head, and mother’s own nutrition status. The latter was measured as body mass index (BMI), using the cut-off point of < 18.5 kg/m^2^ as underweight, 18.5-25 kg/m^2^ as normal nutrition status, 25–30 kg/m^2^ as overweight, and above 30 kg/m^2^ as obese. Wealth index was used as a measure of household economic status by considering households assets ownership and living conditions. The dichotomized variables on such assets were reduced using principle component analyses and item weight assigned to give weighted wealth index which was divided into quintiles into poorest, poorer, middle, richer and richest wealth quintile.

### Data analysis

We conducted descriptive and regression analyses using SPSS Version 22. For the first objective, the descriptive analysis using frequency distributions was done to determine the magnitude of stunting across the six surveys. We used the chi-square test to examine the differences in prevalence of stunting across the years. We also used the chi-square test to assess differences on the characteristics of the caregivers in relation to child stunting across. For the second objective, we used the last survey (2015–2016 TDHS) to examine the factors associated with stunting. This was a mitigation to address the differences in measurements and presentation of independent variables across the surveys and some datasets having significant number of missing variables. For this, we ran a three-level hierarchical logistic regression models to examine factors associated with stunting. Hierarchical logistic regression models are able to avoid distal factors be improperly adjusted by proximate factors [19, 20]. In the first model, we included place of residence as the only distal factor. In the second model we included underlying factors such as care givers characteristics and household characteristics and factors from the first model which had *p*-value< 0.2. In the final (third) model, we included proximal factors like child characteristics and factors from the first and second models which had *p*-value< 0.2. We applied sample weighting generated by the TDHS to adjust for cluster sampling design and sampling probabilities across clusters and strata. A statistically significant level was set at *p* < 0.05.

### Ethical consideration

The use of this data was approved by MEASURE Tanzania Demographic and Health Surveys after our request with the data analysis protocol. During the surveys, the protocols and data collection procedures were approved by relevant authorities in Tanzania mainland and Zanzibar. These include the National Institute of Medical Research (NIMR), Zanzibar Medical Research Ethical Committee (ZAMREC), the Institutional Review Board of ICF International, and the Centers for Disease Control and Prevention in Atlanta. In the data collection procedures, all participants were asked to provide verbal informed consent after the consent statement was read to them which emphasized the voluntary nature of the survey. Interviews were conducted under the private conditions afforded by the environments encountered. Confidentiality was adhered to by making sure that names of respondents were not written in the data collection tools and hence were anonymous.

## Results

### Characteristics of caregivers

Findings revealed a steady improvement in education attainment among caregivers over two and a half decades (Table 1). Over this period, the proportion of caregivers without any formal education had reduced from 35% in 1991–1992 to 21% in 2015–2016 survey. During the same period, caregivers with primary level of education had not significantly changed (62 to 65%) while those with secondary school education increased 3 to 13%. The proportion of women who had a child by 19 years had declined from 69% in 1991 to 62% in 2015 signifying an increase in the age at first birth. Evidence show that caregivers with five or more children declined from 31% in 1991 to 29% in 2015. This is also evidenced by the total fertility rate (TFR) which was reduced from 6.2 in 1991 to 5.2 in 2015.

**Table 1:** Descriptive characteristics of participants in six Tanzania Demographic and Health Surveys (TDHS 1991–2016)

Variables	TDHS 1991–1992		TDHS 1996		TDHS 1999		TDHS 2004–2005		TDHS 2009–2010		TDHS 2015–2016	
	N	%	N	%	N	%	N	%	N	%	N	%
Highest educational level of mother respondents												
No education	2561	35	1812	29	807	28	2085	26	1959	26	2013	21
Primary	4470	62	4155	67	1983	68	5526	69	5219	68	6142	65
Secondary	208	3	207	3	108	4	275	3	468	6	1279	13
Higher	18	0.3	2	0.03			89	1	20	0.3	87	1
Body mass index (kg/m2) of mother respondents												
Low (< 18.50)	638	9	517	9			550	7	681	9	637	7
Normal (18.50–24.99)	5729	80	4715	78			6187	78	5580	74	6476	69
Overweight (25.00–30.00)	698	10	696	12			959	12	1018	13	1616	17
Obese (> 30.00)	123	2	140	2			230	3	300	4	724	8
Age at 1st birth of mother respondents												
0–19	4972	69	4057	66	1951	67	5197	65	4933	64	5922	62
20–29	2252	31	2102	34	939	32	2719	34	2670	35	3483	37
30 and above	32	0.5	28	0.46	8	0.3	59	1	64	1	115	1
Duration of breastfeeding of mother respondents												
< 6	882	12	704	11	347	12	877	11	886	12	982	10
6–12	1334	18	1155	19	505	17	1568	20	1466	19	1128	12
13–24	4234	58	3495	56	1633	56	4609	58	4434	58	1304	14
> 24	702	10	638	10	295	10	709	9	596	8	119	1
Inconsistent breastfeeding	21	0.2	26	0.4	10	0.3	20	0.3	38	0.5	5919	62
Never get breastfeeding	35	0.5	96	2	78	3	162	2	133	2	68	1
Number of living children of mother respondents												
1	1223	17	1057	17	544	19	1318	17	1139	15	1796	19
2	1520	21	1186	19	642	22	1838	23	1627	21	1993	21
3	1241	17	1155	19	481	17	1517	19	1487	19	1716	18
4	993	14	880	14	429	15	1068	13	1118	15	1240	13
5	786	11	666	11	282	10	774	10	852	11	956	10
6–15(> 5)	1493	21	1244	20	520	18	1461	18	1443	19	1819	19
Sex of head of household												
Male	6395	88	5225	84	2415	83	6619	83	6347	83	7875	83
Female	861	12	963	16	484	17	1356	17	1320	17	1645	17
Residence of respondents												
Rural	5808	80	5056	82	2353	81	6417	80	6137	80	6980	73
Urban	1449	20	1132	18	546	19	1558	20	1530	20	2541	27

The magnitude of underweight (measured by low BMI) among caregivers declined from 9% in 1991 to 7% in 2015. The proportion of women with overweight and obesity has more than doubled from 11% in 1991 to 25% just two and a half a decade. In addition, proportion of caregivers who are the breadwinners in their households from 12% in 1991 to 17% in 2015. Majority of the population still resides in rural areas, however, there is a notable decline in the proportion from 80% in 1991 to 73% in 2015.

This is evidenced by a decline in proportion of women with duration of breastfeeding of less than six months from 12% in 1991 to 10% in 2015. However, in 2015 majority of women reported inconsistent breastfeeding practices where there is a notable proportion who had never breastfed.

#### Trend in stunting over two decades

**Fig. 1:**
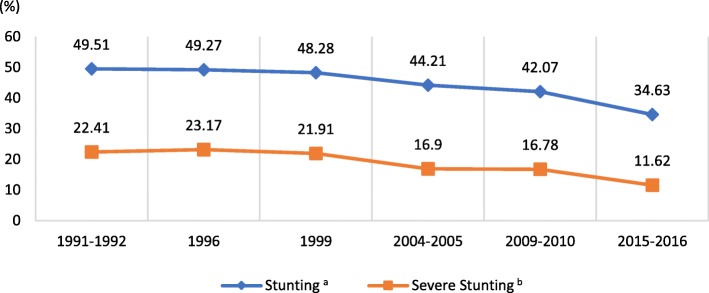
Trends in prevalence of stunting in Tanzania from 1991–2016, TDHS Surveys. ^a^ *P* < 0.001, X^2^ = 523.45. ^b^ *P* < 0.001, X^2^ = 499.11. Blue line: The proportion of children with stunting Tanzania. Orange line: The proportion of children with severe stunting Tanzania. Figure 1 shows the trends in stunting over the two and half decades (1991–2015) in Tanzania. And there were similar trends for prevalence of stunting and severe stunting

Figure 1 shows the trends in stunting over the two and half decades (1991–2015) in Tanzania. The findings show a significant reduction of stunting among children under five. The prevalence of stunting declined from 50% in 1991 to 35% in 2015 (*p* < 0.001). In 1991 one in every two under-fives had stunting compared to one in three under-fives in 2015. The decline is also seen in severe form of stunting which has halved from 22% in 1991 to 12% in 2015.

#### Descriptive characteristics of children in relation to stunting status

A higher proportion of boys have consistently succumbed to stunting compared to their counterparts (*p* < 0.001) **(**Table 2**)**. Moreover, the prevalence of stunting tends to increase as the child grows to its highest proportion during 24–35 months and declines thereafter.

**Table 2:** Stunting prevalence among children under 5 according to the descriptive characteristics in TDHS 1991–2016

variables	1991–1992			1996			1999			2004–2005			2009–2010			2015–2016		
	%	95% C.I.		%	95% C.I.		%	95% C.I.		%	95% C.I.		%	95% C.I.		%	95% C.I.	
Sex of child																		
Male	52	50.69	54.09	52	50.62	54.18	51	48.07	53.59	47	45.29	48.50	41	39.76	43.13	37	35.57	38.39
Female	47	45.00	48.37	46	44.23	47.84	46	42.91	48.46	42	39.93	43.10	43	41.02	44.35	32	30.78	33.57
*P*-value	< 0.001			< 0.001			< 0.001			< 0.001			0.32			< 0.001		
Age of child (months)																		
0–11	30	27.71	32.20	27	24.83	29.51	25	21.91	28.93	26	23.55	27.66	42	39.56	44.68	16	14.55	17.81
12–23	51	48.47	53.47	54	51.02	56.32	53	48.28	56.80	48	46.03	50.89	40	37.48	42.76	38	36.31	40.53
24–35	62	59.20	64.50	62	58.75	64.45	59	55.02	63.63	52	49.41	54.51	43	39.86	45.43	46	44.08	48.85
36–47	59	56.08	61.83	60	56.74	62.66	56	51.55	60.95	53	50.22	55.55	43	39.83	45.29	42	39.10	43.97
48–59	52	48.72	54.61	49	46.11	52.30	53	48.83	58.14	46	43.14	48.68	45	41.61	47.42	33	30.71	35.44
*P*-value	< 0.001			< 0.001			< 0.001			< 0.001			0.34			< 0.001		
Birthweight (g)																		
Low	60	54.91	64.25	63	57.16	68.17	73	64.15	81.85	58	51.43	64.05	40	33.48	46.52	48	42.82	53.41
Normal	46	44.11	47.94	43	41.37	45.34	44	41.01	47.47	41	39.46	42.99	41	39.06	42.67	31	30.09	32.77
High	40	33.81	45.88	41	35.49	47.07	27	19.29	35.11	28	23.27	31.93	40	35.00	44.51	26	23.04	29.68
P-value	< 0.001			< 0.001			< 0.001			< 0.001			0.84			< 0.001		
Body mass index (kg/m^2^) of mother respondents																		
Low (< 18.5)	57	52.98	60.88	54	49.85	58.90				49	44.13	52.89	46	41.62	49.44	39	35.29	43.11
Normal (18.5–24.9)	50	48.61	51.31	51	49.40	52.29				46	45.05	47.61	42	40.46	43.23	37	35.39	37.81
Overweight (25–30)	41	37.40	45.00	38	34.43	41.81				32	28.91	35.14	42	38.83	45.41	31	28.93	33.70
Obese (> 30)	41	29.90	49.31	38	29.50	46.47				32	17.12	28.88	42	36.94	49.35	31	16.80	23.01
*P*-value	< 0.001			< 0.001						< 0.001			0.56			< 0.001		
Age of mother respondents at 1st birth																		
0–19	51	50.02	52.92	50	48.52	51.64	48	46.03	50.81	45	43.39	46.19	42	40.03	42.97	35	33.72	36.25
20–29	45	43.10	47.41	48	45.33	49.75	48	44.50	51.35	43	41.22	45.09	43	41.06	45.10	34	32.58	35.86
30 and above	42	23.54	60.33	58	37.34	78.04	57	7.71	106.58	41	27.20	54.28	46	30.70	60.61	28	19.59	37.02
*P*-value	< 0.001			0.33			0.76			0.43			0.53			0.13		
Time after the birth at which the mother respondent first breastfed the last child																		
Immediately	48	46.30	50.65	45	43.08	47.19				42	40.55	43.71	39	37.23	41.17	34	32.85	35.82
Within 1 day	45	43.04	47.58	50	46.92	52.16				46	44.10	47.69	43	41.75	44.93	35	33.05	36.08
Over 1 day	47	42.82	50.26	48	43.22	52.19				48	44.06	52.27	46	41.08	50.22	35	30.25	39.10
*P*-value	0.20			0.14						< 0.001			0.01			0.90		
Duration of breastfeeding (months)																		
< 6	26	22.89	28.84	22	18.98	25.15	23	17.96	27.26	21	18.54	24.07	39	35.59	42.53	13	10.75	15.01
6–12	41	37.80	43.22	40	36.71	42.43	37	32.16	41.24	35	32.89	37.72	43	40.38	45.80	19	16.92	21.54
13–24	55	53.62	56.76	55	53.05	56.43	54	51.55	56.80	50	48.03	51.05	42	40.10	43.22	38	35.28	40.60
> 24	64	60.33	67.83	68	63.97	71.48	66	60.05	71.53	60	55.80	63.20	48	43.38	51.82	52	42.86	61.56
Inconsistent breastfeeding	24	1.05	46.01	19	2.86	34.18	33	−20.86	87.53	33	9.21	57.46	39	21.80	56.99	41	39.32	41.97
Never get breastfeeding	66	49.69	83.64	57	47.27	67.63	42	29.23	53.84	46	38.18	54.78	43	34.61	53.11	34	20.78	47.14
*P*-value	< 0.001			< 0.001			< 0.001			< 0.001			0.13			< 0.001		
Number of births in last five years																		
1	52	49.56	53.63	52	49.84	53.91	47	44.12	50.41	45	42.86	46.52	41	39.46	43.23	33	31.87	34.77
2	49	47.27	50.52	48	46.55	50.16	50	47.21	52.87	45	43.18	46.43	43	40.82	44.21	37	35.11	38.13
3	45	41.07	48.66	44	40.22	47.97	45	39.53	50.37	41	37.76	44.17	43	39.57	46.77	33	29.36	35.94
> 3	58	44.68	71.11	48	31.87	63.37	43	−6.58	92.29	35	23.64	46.95	35	20.48	49.08	18	7.66	28.70
*P*-value	0.01			0.02			0.35			0.10			0.35			0.00		
Number of living children																		
1	48	45.51	51.46	50	47.28	53.39	44	39.36	48.47	45	42.21	47.85	41	37.61	43.85	32	29.99	34.48
2	47	44.69	50.06	49	46.19	52.09	41	36.96	45.24	44	42.01	46.81	41	38.69	43.79	35	32.30	36.71
3	49	45.76	51.59	48	44.65	50.55	48	42.85	52.41	45	42.70	47.91	42	38.87	44.20	31	29.10	33.73
4	50	46.50	52.87	49	45.28	52.01	55	49.49	59.65	43	40.26	46.35	41	38.38	44.58	35	32.19	37.68
5	53	49.38	56.63	50	45.98	53.71	48	41.86	54.46	44	40.05	47.28	43	39.69	46.83	38	34.45	40.83
6–15	51	48.46	53.63	50	47.30	52.95	57	52.18	61.24	43	40.56	45.69	44	41.62	47.11	38	35.87	40.44
*P*-value	0.21			0.83			< 0.001			0.94			0.77			< 0.01		
Highest educational level																		
No education	51	49.36	53.38	54	52.10	56.83	50	46.54	53.88	47	44.81	49.27	42	39.70	44.34	39	37.25	41.71
Primary	49	47.49	50.54	48	46.44	49.50	48	46.08	50.80	44	42.72	45.41	43	41.08	43.94	35	33.98	36.44
Secondary	34	26.70	41.65	26	18.67	33.51	23	12.56	33.60	33	26.09	39.24	36	30.76	41.05	25	22.49	27.65
Higher	53	27.91	77.36							10	2.75	16.97	53	24.74	81.93	8	1.74	14.48
*P*-value	< 0.001			< 0.001			< 0.001			< 0.001			0.02			< 0.001		
Sex of head of household																		
Male	49	47.78	50.33	49	47.67	50.42	47	45.36	49.62	43	41.84	44.30	42	40.57	43.16	34	32.85	35.02
Female	53	49.54	56.67	51	47.25	53.89	53	47.59	57.46	50	47.12	52.71	43	40.20	46.00	38	35.57	40.51
*P*-value	0.08			0.64			0.07			< 0.001			0.62			0.01		
Number of household members																		
1–5	51	48.88	53.21	50	47.65	51.89	51	47.52	54.00	46	44.11	47.72	42	40.11	43.92	35	33.30	36.48
6–10	51	48.88	52.30	49	47.41	50.96	49	45.70	51.64	45	43.31	46.61	42	40.53	44.02	35	33.79	36.71
> 10	44	41.81	47.16	48	44.64	51.78	43	38.49	47.22	37	33.74	39.67	42	38.56	44.67	32	29.11	34.36
*P*-value	< 0.001			1			0.02			< 0.001			0.89			0.13		
Residence of household																		
Rural	50	48.80	51.45	51	49.85	52.65	52	50.01	54.30	46	45.04	47.55	43	41.23	43.87	38	36.65	39.00
Urban	47	43.90	49.54	40	36.99	42.93	29	24.47	33.22	35	32.36	37.43	40	37.43	42.78	25	23.59	27.18
*P*-value	< 0.01			< 0.001			< 0.001			< 0.001			0.10			< 0.001		

Children with low birthweight have consistently high prevalence of stunting compared to those born with normal or high birthweight. In 1991, about 60% children born with a low birthweight had stunting compared to 46 and 40% of normal and high birth weight respectively (*p* < 0.001). In 2015, 48% of low birth weight children became stunted compared to 31% of those born with normal weight (*p* < 0.001). Mothers’ nutrition was also found to be a predictor of children’s stunting status. Children of mothers with low BMI were more likely to suffer from stunting compared to their counterparts whose mothers had normal BMI, or even those with overweight and obesity (*p* < 0.001). Mothers’ age at first birth showed mixed results over all surveys with inclination to higher magnitude of stunting when the age was 19 years and below in 1991 (*p* < 0.001), and others but with no statistically significant level.

With regards to feeding characteristics, children who were breastfed immediately had low magnitude of stunting, but the results were significant only in 2004 and 2009. Counter intuitively, breastfeeding duration was not protective for stunting, but those who were never breastfed were more likely to suffer from stunting compared to even the children who were breastfed for a shorter period of time. Moreover, women who had more than one birth in the past five years were less likely to have their children stunted compared to those with one birth. However, the more the number of children living in a household increases the risk of stunting increased as well.

Although the proportion of women-headed household showed to increase, the findings show that, the female lead households were more likely to have a stunted child compared to the one led by a male. In two surveys, this association is statistically significant. Findings further show that the higher the number of household’s members the lower the risk of stunting. The prevalence of stunting is also consistently higher among children in rural households compared to their urban counterparts (*p* < 0.001).

#### Factors associated with stunting in Tanzania

Table 3 shows the results of three-level hierarchical logistic regression analyses. Children living in urban areas were less likely to be stunted compared to their counterparts living in rural area (AOR = 0.56, 95%CI = 0.50–0.62, *P* < 0.001). There was no statistically significant association between mothers’ age at first birth with child stunting status. However, mothers’ education was protective against child’s stunting. Children whose mothers had higher education were 44% less likely to be stunted compared to those whose mothers had no education (*P* = 0.018). Children whose mothers were obese were less likely to suffer from stunting compared to their counterparts whose mothers had normal BMI (AOR = 0.63, 95%CI = 0.51–0.78, *P* < 0.001). After adjusting for possible confounders, children from households lead by females (AOR = 1.16, 95% CI = 1.03–1.31, *P* = 0.014) were more likely to be stunted compared to the male lead households. Wealthier households were less likely to have stunted children. For example, children in the two higher wealth quintiles were 35 and 58% less likely to be stunted compared to poorest children (*P* < 0.001).

**Table 3:** Factors associated with stunting using TDHS 2015–2016

Variable	N (%)	Model 1^a^			Model 2^b^			Model 3^c^		
		AOR	95% CI	*p*-value	AOR	95% CI	*p*-value	AOR	95% CI	*p*-value
Residence										
Rural	6531 (74)	Reference								
Urban	2284 (26)	0.56	(0.50, 0.62)	< 0.001						
Maternal age in years										
15–19	596(7)				Reference					
20–24	2064(23)				1.31	(1.06, 1.62)	0.014			
25–29	2205(25)				1.08	(0.84, 1.39)	0.540			
30–34	1693(19)				1.07	(0.8, 1.42)	0.662			
35–39	1339(15)				1.08	(0.78, 1.49)	0.634			
40–44	731(8)				1.23	(0.86, 1.75)	0.248			
45–49	186(2)				1.70	(1.09, 2.65)	0.019			
Mother’s body mass index (kg/m2)										
< 18.5	605(7)				1.09	(0.92, 1.3)	0.328			
18.5–24.9	6034(68)				Reference					
25.0–30.0	1478 (17)				0.85	(0.75, 0.97)	0.015			
> 30.0	676 (8)				0.63	(0.51, 0.78)	< 0.001			
Mother’s age at 1st birth										
< 20	5451(62)				0.94	(0.84, 1.05)	0.282			
20–29	3256(37)				Reference					
30>	107(1)				0.99	(0.62, 1.59)	0.976			
Number of births in last five years										
1	4014(46)				Reference					
2	3917(44)				0.99	(0.88, 1.11)	0.867			
3	820(9)				0.85	(0.7, 1.03)	0.090			
> 3	64(1)				0.48	(0.26, 0.91)	0.024			
Number of living children										
1	1637 (19)				Reference					
2	1793 (20)				1.07	(0.89, 1.28)	0.455			
3	1575 (18)				1.00	(0.81, 1.24)	0.993			
4	1155(13)				1.11	(0.86, 1.42)	0.429			
5	899(10)				1.17	(0.88, 1.56)	0.267			
6–15	1755(20)				1.17	(0.86, 1.59)	0.325			
Highest educational level										
No education	1891 (21)				Reference					
Primary	5686 (65)				0.99	(0.88, 1.1)	0.830			
Secondary	1161(13)				0.83	(0.63, 1.06)	0.305			
Higher	76(1)				0.56	(0.16, 0.91)	0.018			
Head of household										
Male	7348(83)				Reference					
Female	1467(17)				1.16	(1.03, 1.31)	0.014			
Number of household members										
1–5	3429 (39)				Reference					
6–10	4137 (47)				0.88	(0.79, 0.99)	0.027			
> 10	1248(14)				0.75	(0.65, 0.87)	0.000			
Wealth index										
Poorest	2009(23)				Reference					
Poorer	1932(22)				0.96	(0.85, 1.09)	0.563			
Middle	1778(20)				0.98	(0.86, 1.12)	0.758			
Richer	1818(21)				0.65	(0.56, 0.76)	< 0.001			
Richest	1278(14)				0.42	(0.34, 0.52)	< 0.001			
Age of child (months)										
0–11	1970(22)							Reference		
12–23	2055(23)							1.50	(0.97, 2.32)	0.069
24–35	1688(19)							1.75	(1.09, 2.79)	0.019
36–47	1580(18)							1.38	(0.86, 2.21)	0.181
48–59	1521(17)							0.99	(0.62, 1.59)	0.967
Sex of child										
male	4469(51)							Reference		
female	4345(49)							0.77	(0.68, 0.87)	< 0.001
Birthweight (g)										
low < 2500	340 (6)							2.14	(1.68, 2.71)	< 0.001
4000 > normal> = 2500	4551(82)							Reference		
high > = 5000	677 (12)							0.65	(0.54, 0.79)	< 0.001
Duration of breastfeeding (months)										
< 6	962 (11)							Reference		
6–12	1114(13)							1.63	(1.18, 2.24)	0.003
13–24	1281(15)							2.91	(1.73, 4.91)	< 0.001
> 24	111(1)							5.44	(2.57, 11.49)	< 0.001
Inconsistent breastfeeding	5288(60)							3.46	(2.07, 5.78)	< 0.001
Never breastfed	58(1)							4.29	(1.77, 10.37)	0.001

*N (%)* N frequency; (%) percentage of frequency, *AOR* Adjusted Odds Ratio, *C.I.* Confidence interval

^a^Model-1: adjusted for residence place

^b^Model-2: adjusted for residence place and all variables shown under Model-2

^c^Model-3: adjusted for residence place and all variables shown under Model-3

Children aged 24–35 months had higher risk of stunting compared to those of 0–11 months (AOR = 1.75, 95% CI = 1.09–2.79, *P* = 0.019). Girls were less likely to be stunted compared to boys (AOR = 0.77, 95%CI = 0.68–0.87, *P* < 0.001). Compared with under-fives born with normal birth weight, those born with lower birth weights were twice more likely to be stunted (*P* < 0.001), while children born with higher birth weight were 35% less likely to be stunted (*P* < 0.001).

Compared to children who were breastfed for less than six months, those who were inconsistent breastfeeding or never breastfed were more likely to be stunted (AOR = 3.46, 95% CI = 2.07–5.78, *P* < 0.001 and AOR = 4.29, 95% CI =1.77–10.37, *P* < 0.001 respectively).

## Discussion

Evidence from the secondary analyses of Tanzania Demographic Surveys conducted from 1991 to 2016 suggest a steady decline of stunting among under-five children. There is also a notable improvement in women’s empowerment that could have influenced the changes. This is shown through a notably steady improvement in education attainment among caregivers, increase in the age at first child birth, and proportion of female heading households. Such progress made may have influenced better nutritional status among mothers and subsequently consistent decline in the burden of low birthweight among children as one of the pathways towards stunting reduction [21].

The magnitude of undernutrition and in particular stunting is on a steady decline globally but with slow pace among sub Saharan African countries [16, 22]. Tanzania is no exception [4]. This chronic form of undernutrition was prevalent among 49.5% of children under five in 1991, translated to one in every two under-fives [4]. Twenty five years later, and with efforts and investment in health and human capital development in the country, the magnitude of stunting has declined to 34% in 2015–2016 [4, 16], translated to one in every three under-fives. Although this 30% decline is significant over two and a half decades, the prevalence of stunting at this level is one of the highest globally [16]. Tanzania remains one of the 14 countries with the 80% of global burden of stunting.

Evidence from this and previous studies suggest that efforts to ameliorate stunting should also focus in tailored nutrition specific and sensitive interventions [23]. Owing to diversity of causes of undernutrition, there is no silver bullet for stunting. To this end, a combination of such nutritional sensitive and specific interventions tailored to the local context can make impact [24, 25]. In the context of Tanzania, socio-demographic disadvantaged populations bear a significant brunt of stunting like other forms of undernutrition [4, 26, 27]. Like in such previous studies, this analysis found that, the risk of stunting increases with poverty and low educational attainment of caregivers. Poverty renders populations into poor health services and poor feeding practices. Evidence further suggests a closer link between the mentioned basic and underlying causes or determinants with immediate causes such as poor feeding practices. Our current analyses also confirmed an association between poor IYCF practices with stunting. Moreover, stunting was higher among children aged 24 months and above compared to those under one year. The peak age (24–35 months) has a significant nutritional milestone where the child is mostly independent, finished breastfeeding, and fully introduced to normal foods as per a specific place.

With improvement of the pertinent determinants like in the context of Tanzania, the indicators for stunting and other forms of undernutrition are improving. The slow pace is due to difficulty in addressing underlying and basic causes of undernutrition that call for nutrition sensitive approaches. As evidenced elsewhere, improvement of population-wide stunting may take longer than other forms of acute forms of undernutrition whose determinants are immediate causes such as poor feeding practices and disease conditions and have specific interventions that are easily attained [4, 16].

This study reveals an increase in proportion of women who lead their households. However, data suggests that when a woman leads the household the children’s nutrition status is not improving. Tanzania is composed of mostly patrilineal societies where majority of households are led by male figures. When a woman leads the households, she may likely be a single mother, widowed, or living alone. These living arrangements translate to low household income from a single adult compared to others where both mother and father may be contributing to household wealth. It also is assumed that, the increase in the number of female household leaders may be due to improved education level, self-sufficiency, and engagement in direct economic activities with a results of better feeding practices. This may also affect the children as their only parent would be spending more time seeking for means to sustain the household than taking care of the children nutritional needs as in traditional households in Tanzania. Although to an extension, it is a product of higher educational attainment, those with the highest education levels tend to have less advantage in stunting reduction owing to less time they have for child caring. Efforts should therefore be streamlined to working mothers, including those with higher income.

Results from this analysis further emphasize on the interventions in the first 1000 days of life [13, 28, 29]. Both maternal and newborn health are important to address future nutrition status of children [30]. This study found a declining burden of maternal undernutrition and low birthweight. Nevertheless, such important determinants are still persistent and remain behind the current high burden of stunting. Tanzania is also facing a nutritional transition challenge owing to increased burden of overweight and obesity amid persistent burden of undernutrition [4, 16]. Such poor magnitude of overweight and obesity more than doubled between 1991 and 2015 [4]. This call for renewed efforts to address double edged sword of undernutrition and overweight and obesity that will continue to burden health system in Tanzania through non-communicable diseases.

Evidence in this study puts emphasis on importance of addressing stunting among boys. This evidence is not strange in the context of sub Saharan Africa [31]. A number of explanations have been given including biologically, socially, and feeding differences among girls as compared to boys [31].

Evidence from this study should be interpreted carefully owing to the following two limitations. First, the analysis was based on cross sectional surveys that does not provide causal pathways even though a number of serial cross sectional studies were analyzed together. Findings from this study, however, are not different from other well-designed studies and further strengthen the existing strong-designed studies. Second, data were collected across years with various improvements in the data collection tools and methods. In TDHS surveys, while procedures of data collection remained the same, some variables have changed or modified to address the new needs. An example for this is dietary diversity scales, and nutritional assessment reference populations. We dropped the dietary diversity data owing to significant differences between years and we used one standardized population (the WHO standards) for nutritional status. Despite the two limitations, this is the first study that analyzed data across all national representative surveys for the past two and half a decade in Tanzania. Evidence presented here is based on a big sample size making it easy to generalize the findings for Tanzania as well and with power to generate conclusions.

## Conclusions

In conclusions, Tanzania is making progress in addressing the burden stunting among children under five. This comes about through strides made to improve socio-demographic challenges including women empowerment, inclusiveness in education and contribution to the national economy for women. The pace for ameliorating stunting, however, is slow—with remaining burden among one in every three under-fives. This unprecedented burden calls for the multisectoral collaboration efforts to address the remaining determinants of stunting which include low birth weights, poor nutritional status of mothers, short duration of breastfeeding and low wealth status of the families. Effective tailored nutritional sensitive interventions should be thought thoroughly to address these significant determinants.

## Data Availability

All datasets are available upon request from DHS website.

## Abbreviations

AIS: AIDS Indicator Surveys
KIS: Key Indicator Surveys
MIS: Malaria Indicator Surveys
SPA: Service Provision Assessment
AOR: Adjusted Odds Ratio
CDC: Center for Disease Control
MEASURE: Monitoring and Evaluation to Assess and Use Results
NBS: National Bureau of Statistics
NCHS: National Center for Health Statistics
NIMR: National Institute of Medical Research
TDHS: Tanzania Demographic and Health Survey
TFR: Total Fertility Rate
WHO: World Health Organization
ZAMREC: Zanzibar Medical Research and Ethics Committee
